# The Persian Version of Utrecht Questionnaire for Evaluation of Aesthetic Rhinoplasty Outcomes: Translation and Validation

**DOI:** 10.29252/wjps.9.2.141

**Published:** 2020-05

**Authors:** Hesam Jahandideh, Fatemeh Dehghani Firouzabadi, Mohammad Dehghani Firouzabadi, Peter JFM Lohuis, Maryam Roomiani

**Affiliations:** 1 Department of Otolaryngology, ENT and Head and Neck Research Center, Firoozgar Hospital, Iran University of Medical Sciences, Tehran, Iran;; 2 ENT and Head and Neck Research Center and Department, Five Senses Health Research Institute, Iran University of Medical Sciences, Tehran, Iran;; 3 Department of Otolaryngology, University Hospital Svet Duh, Zagreb, Kroatië;; 4 Department of Otolaryngology, Lohuis-filipovic Medical Group, Zagreb, Kroatië

**Keywords:** Utrecht questionnaire, Rhinoplasty, Validation, Personal satisfaction, Quality of life

## Abstract

**BACKGROUND:**

There are different questionnaires and approaches to evaluate the outcome of rhinoplasty operations. A short questionnaire, which can be completed in less than 2 minutes, is the Utrecht questionnaire that consists of a visual analogue scale (VAS) and five multiple-choice questions. In this study, we have translated the questionnaire in Persian and evaluated its reliability and validity.

**METHODS:**

Patients undergoing aesthetic rhinoplasty surgery in Firoozgar Hospital from January to March 2019 were enrolled. The questionnaire was translated to Persian and backward translated to English by independent medical extern Persian speakers with complete English proficiency. The internal consistency was measured by Cronbach’s alpha, repeatability by Student *t* test of test-retest 4 weeks and 12 weeks follow-up post-operatively, and validity by comparing pre- and post- operative results.

**RESULTS:**

Thirty patients were included in the analysis. The Cronbach’s alpha was 0.925 as a marker for internal consistency. The test-retest was acceptable for all the questions accordingly (*p*>0.05). The p values for pre- vs. post-operative tests were also significant for either all of the questions and the sum score.

**CONCLUSION:**

The translated questionnaire was internally consistent and repeatable. The questionnaire also seems to be valid for all questions and the sum score. According to our analysis, the translated Persian version of the Utrecht questionnaire seems to be internally consistence, reliable in test-retest analysis, and valid due to a pre-post operational analysis.

## INTRODUCTION

Aesthetic rhinoplasty surgery is performed recently more than the past.^1^ It is important to measure patients’ satisfaction postoperatively. There is a considerable need for a rapid reliable questionnaire in order to evaluate this aesthetic surgery’s outcome in patients’ point of view.^2^ Also, this questionnaire seems to be crucial for evaluating the approaches and the patient’s satisfactory level with attention to developing more surgery approaches and methods.^3^ Various questionnaires have been used since now for this purpose and wide range of studies have discussed their advantages and disadvantages.^4^

Using Facial Appearance Sorting Test, Glasgow Benefit Inventory, and quality of life questionnaires; they are supposed to be some of the applicable approaches for the evaluation.^2^^,^^5^ On the other hand, Lohuis *et al.* suggested a questionnaire, called Utrecht questionnaire (UQ), which can not only evaluate interior body image and body image, but also can be completed within 2 minutes, as they believed that pervious questionnaires seem to be long and patients may forget some points.^2^ UQ consists of two parts as the first one is a visual analogue scale (VAS) that can determine patient’s perception about his appearance (0 as very ugly and 10 for very nice).

The second part includes five multiple-choice questions that can identify patient’s quality of life with respect to nasal appearance, every question was graded in a five-point Likert scale (from 1, not at all to 5, very much/often).^1^^,^^2^ The questionnaire was translated and validated in different languages^1^^,^^6^ and our aim was to measure the reliability and validity of its Persian translated version.

## MATERIALS AND METHODS

Two English to Persian translators who were familiar with medical terms translated the questionnaire as the early Persian version. They were asked to prepare a translation for general population and using simple words, instead of professional terms. The two early Persian versions were evaluated by ENT specialists’ panel and the result was backward translated to English by a Persian to English translator who was not aware of the main English version. Considering the required revisions, the final questionnaire was used as shown in Table 1. 

**Table 1:** Original version of the Utrecht Questionnaire for Outcome Assessment in Aesthetic Rhinoplasty with the corresponding translation in Iran language

**I give the following score to the way I like the appearance of my nose:**					
**0---1---2---3---4---5---6---7---8---9---10**					
**Very ugly**			**Very nice**		
E1. Are you concerned about the appearance of your nose?					
Not at all	A little	Moderate		Much or often	Very much/often
E2. Dose this concern bother you often?					
Not at all	A little	Moderate		Much or often	Very much/often
E3. Does this concern affect your daily life? (e.g., your work)					
Not at all	A little	Moderate		Much or often	Very much/often
E4. Does this concern affect your relationships with others?					
Not at all	A little	Moderate		Much or often	Very much/often
E5. Do you feel stressed by the appearance of your nose?					
Not at all	A little	Moderate		Much or often	Very much/often

All patients undergoing aesthetic rhinoplasty from January to March 2019 in Firoozgar Hospital, Tehran, Iran were asked to complete the questionnaire pre-operatively and 4 weeks, and 12 weeks post-operatively. The exclusion criteria were solitary septoplasty and some associated surgeries, such as sinus surgery as well as congenital facial anomalies, who did not speak Persian. Informed consent was obtained from each individual. The study protocol conformed to the ethical guidelines of the 1975 Declaration of Helsinki as reflected by a priori approval by the institution’s Human Research Committee.

Internal consistency was evaluated through Cronbach’s alpha test. According to Clark and Watson, inter-item and total-correlations above 0.7 was considered to be acceptable, above 0.8 was defined as good, and above 0.9 was considered as excellent.^7^ The repeatability was also assessed by Wilcoxon test, due to their distribution, between 4-week and 12-week tests. A Wilcoxon test was also used in order to compare pre- vs. post-operative results to evaluate the validity of the questionnaire. As nose satisfaction was a subjective parameter, related to patients’ emotions, the questionnaire was considered to be valid, if the patients’ satisfaction improved after a standard aesthetic surgery. All tests were performed using IBM SPSS Statistics software (Version 25.0, Chicago, IL, USA). *p*<0.05 was considered statistically significant. 

## RESULTS

Totally, 48 patients completed our questionnaires before the operation, and 4 weeks and 12 weeks after that. The Cronbach’s alpha for the tests were 0.925, which were considered adequate internal consistency.^7^ Removing any question would yield to lower Cronbach’s alpha. Summary of the Cronbach’s alpha measurement was demonstrated in Table 2. The comparison between 4-week and 12-week tests can be seen in Table 3. The *p *value of the Wilcoxon was greater than 0.05 for all the questions which suggested repeatability of the translated questionnaire (Table 3).

**Table 2:** Cronbach’s alpha measures for questions, individually

**Question in English**	**Corrected item-total correlation**	**Cronbach’s alpha if item deleted**
E1	0.529	0.819
E2	0.766	0.746
E3	0.617	0.792
E4	0.560	0.807
E5	0.643	0.783

**Table 3:** Comparing the 4-weeks and 12-weeks follow-up results

**Question**	**4 weeks mean (SD)**	**12 weeks mean (SD)**	***p*** ** value**
VAS	7.49 (0.649)	7.57 (0.890)	0.414
E1	1.73 (0.446)	1.65 (0.561)	0.346
E2	1.43 (0.500)	1.41 (0.497)	0.827
E3	1.33 (0.474)	1.24 (0.522)	0.206
E4	1.29 (0.456)	1.18 (0.391)	.197
E5	1.29 (0.456)	1.22 (0.422)	0.467
E1 to E5 sum	7.06 (1.725)	6.71 (1.414)	0.139

Comparison of pre-operative scores with 4-week and 12-week tests can be seen in Table 4. All the parameters individually and the total score in pre-operative test were significantly lower than 4-week and 12-week post-operative tests; which are in accordance with our expectation from aesthetic rhinoplasty surgeries.

**Table 4:** Comparing pre- vs. post-operative results of the questionnaires

**Question**	**Pre-operative mean (SD)**	**4 weeks post-operative mean (SD)**	***p*** ** value** ^a^	**12 weeks** **post-operative mean (SD)**	***p*** ** value** ^a^
VAS	6.65 (1.329)	7.49 (0.649)	<0.001	7.57 (0.890)	<0.001
E1	2.24 (0.830)	1.73 (0.446)	<0.001	1.65 (0.561)	<0.001
E2	1.98 (0.750)	1.43 (0.500)	<0.001	1.41 (0.497)	<0.001
E3	1.61 (0.671)	1.33 (0.474)	<0.001	1.24 (0.522)	0.001
E4.	1.76 (0.830)	1.29 (0.456)	<0.001	1.18 (0.391)	<0.001
E5	1.80 (0.816)	1.29 (0.456)	<0.001	1.22 (0.422)	<0.001
E1 to E5 sum	9.39 (2.992)	7.06 (1.725)	<0.001	6.71 (1.414)	<0.001

a: According to Wilcoxon test

## DISCUSSION

The major popular aesthetic surgery in Iran is rhinoplasty as Iran is named the capital of rhinoplasty in the world.^8^ It accounts for the highest rate of surgery in women due to the prominence of their nose in Islamic dress code in our country.^9^ By improving the beauty of the face, quality of life in patients would be raised. Previous studies showed improving quality of life in patients after 6 months of rhinoplasty.^10^ We assigned that it happens even one month after rhinoplasty, as well. Patient satisfaction and quality of life are literally the most important outcomes of rhinoplasty in not only women, but also men.^11^

Most of them also repeat rhinoplasty for the second and third time to improve their beauty and to enhance their self-confidence, as well as raising the social life.^11^ Therefore, it is important for otorhinolaryngologist and plastic surgeons to measure patients’ point of view before the surgery. Utrecht is a short, useful questionnaire for determining postoperative patient satisfaction about aesthetic rhinoplasty before operation.^8^ This questionnaire should be not only convenient, but also unchangeable based on different translations and cultures as mention before by previous studies.^2^^,^^6^

In this study, we translated the questionnaire in Persian and evaluated its validity and reliability. Our study measured test-retest reliability for every question by using Wilcoxon correlation coefficient among preoperative and postoperative (after 4 and 12 weeks) results. Longer follow ups can assure the repeatability of the questionnaire even more. On the other hand, satisfaction improvement might be affected by getting used to the new appearance as patients may be stressed in the first weeks about the outcome, but not after one month. Doing test-retest analysis might be beneficial as difference in test-retest analysis was reported before as German version, but was not statistically significant.^1^

However, the test-retest analysis was also acceptable for our study within 4 to 12 weeks post-operation, but minor variations through the time is not far from expectation. As Cronbach’s alpha measurement suggests, our Persian version had an adequate internal consistency (alpha=0.925). It is comparable with its Portuguese (0.86) and German (0.91) version. Comparing pre- vs. post-operative evaluations, as a measure of validity, was definitely significant after both 4 and 12 weeks. However, considering the fifth questions focussed on feeling stressed (do you feel stressed with the appearance of your nose?).

There are too many psychological and cultural factors that may contribute to this question, which has also mentioned earlier.^12^ It may dilute the effect of rhinoplasty on patients’ feelings. This study detected strong significant difference between pre-operational and post-operational feeling of stress. However, the fifth question should be analyzed with caution in collectivist societies. Here, the Persian version of the Utrecht questionnaire was evaluated and seems to have acceptable internal consistency, beside acceptable repeatability, and considerable validity. Larger studies would be useful to evaluate the effect of time on patients’ self-evaluation. However, the Persian version of the Utrecht questionnaire seems to be valuable and useful for a variety of researches, such as evaluation of aesthetic nose plastic surgery. 
